# New species of the genus *Inversidens* Haas, 1911 (Unionoida, Unionidae, Gonideinae) from Jiangxi Province, China

**DOI:** 10.3897/zookeys.1054.69075

**Published:** 2021-08-03

**Authors:** Ruiwen Wu, Xiongjun Liu, Takaki Kondo, Shan Ouyang, Xiaoping Wu

**Affiliations:** 1 School of Life Science, Shanxi Normal University, Linfen 041000, China Shanxi Normal University Linfen China; 2 School of Life Science, Jiaying University, Meizhou 514015, China Jiaying University Meizhou China; 3 Division of Natural Science, Osaka Kyoiku University, Osaka 582-8582, Japan Osaka Kyoiku University Osaka Japan; 4 School of Life Sciences, Nanchang University, Nanchang 330031, China Nanchang University Nanchang China

**Keywords:** COI, freshwater mussel, genetic distances, morphology, taxonomy

## Abstract

We diagnose and describe a new freshwater mussel species of the genus *Inversidens*, *I.rentianensis***sp. nov.** from Jiangxi Province, China based on morphological characters and molecular data. This paper includes a morphological description and photograph of the holotype, and partial sequences of mitochondrial COI as DNA barcode data.

## Introduction

The genus *Inversidens* Haas, 1911 belongs to the subfamily Gonideinae in the family Unionidae. The genus was first depicted by Haas (1911) as a subgenus of *Nodularia* with two species, i.e., *Uniobrandtii* Kobelt, 1879 and *Nodulariaparcedentata* Haas, 1911, both restricted to Japan. Later, Haas (1969) further classified *Unioreinianus* Kobelt, 1879, *Uniohaconensis* Ihering, 1893, *Uniojapanensis* Lea, 1859, *Uniopantoensis* Neumayr, 1899 within *Inversidens*. All species were restricted to Japan, except for *U.pantoensis*, which was distributed in China. By comparing the conchological characters, Kondo (1982) believed that *U.brandtii* was not morphologically distinct from *N.parcedentata*, and regarded *N.parcedentata* as a variety of *U.brandtii*. Based on the morphology of the glochidium, Habe (1991) removed *U.reinianus* from *Inversidens*, and established a new genus *Inversiunio*. Based on morphological characteristics of the shell, Kondo (1998) moved *Uniohaconensis*, which was regarded as a synonym of *Uniojokohamensis* (Ihering, 1893), into *Inversiunio*. Furthermore, Starobogatov (1970) used *Uniojapanensis* as the type species for his newly established genus *Pronodularia*.

**Figure 1.**
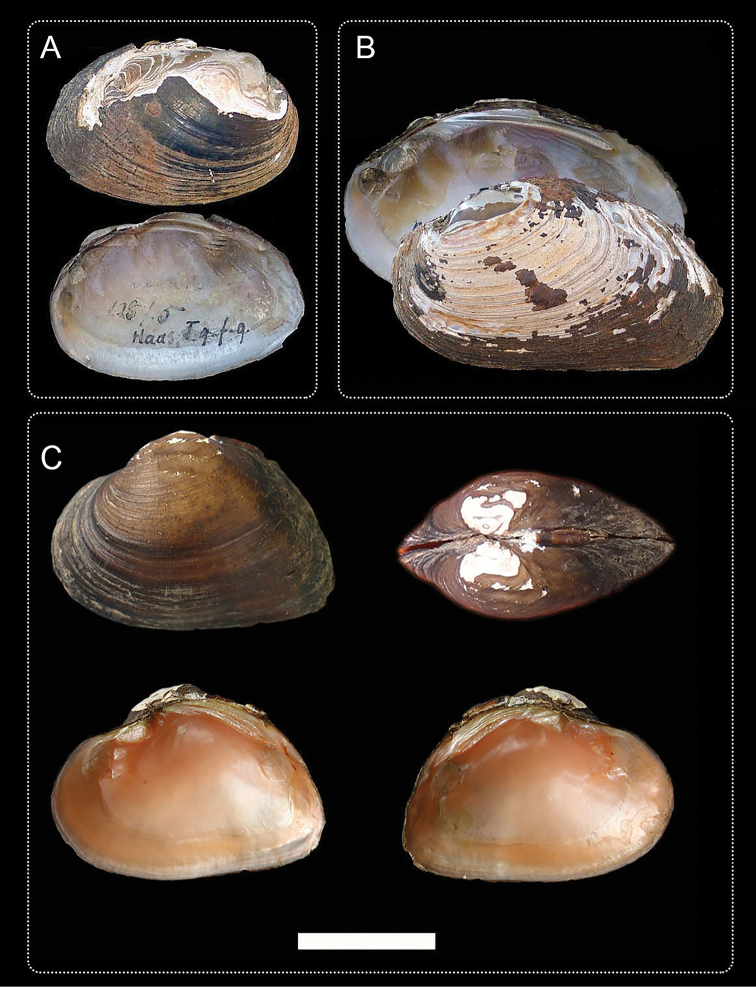
Photographs of *Inversidens* taxa **A***I.brandtii***B***I.pantoensis***C***I.rentianensis* sp. nov. Photos: [**A, B**] from the MUSSEL Project, [**C**] from this study, NCFM180325 (holotype), scale is 2 cm.

Currently, only two species are recognized within *Inversidens*, the Japanese endemic *I.brandtii* and *I.pantoensis* in China (Fig. 1A, B; Kondo 2008; He and Zhuang 2013; Lopes-Lima et al. 2020; Graf and Cummings 2021a, b; MolluscaBase eds. 2021).

In this study, we diagnose and describe a new *Inversidens* species from Jiangxi Province, China. In addition, we provide estimations of the intraspecific and interspecific genetic distances within *Inversidens* based on the mitochondrial COI barcode to examine species validity.

## Materials and methods

### Specimen collection and identification

In March 2018, four samples were collected from the Mianshui River, Rentian Town, Ganzhou City, Jiangxi Province, China (25.989557°N, 116.131333°E). All type and voucher specimens are deposited at the Biological Museum of Nanchang University, China (NCFM180325-NCFM180328).

### DNA extraction and COI amplification

Of the four individuals, only two samples had tissues. Total genomic DNA was extracted from dissected somatic tissue using TIANamp Marine Animals DNA Kit (Tiangen Biotech, Beijing, China) according to the manufacturer’s instructions.

Mitochondrial cytochrome oxidase subunit I (COI) gene sequences have been widely used for species delimitation of freshwater mussels based on genetic distance and the criteria of monophyly (Elderkin et al. 2016; Lopes-Lima et al. 2019; Smith et al. 2019). Polymerase chain reaction (PCR) amplification of the COI gene with a 680-base pair fragment was performed using a primer pair consisting of LCO1490 and HCO2198 (Folmer et al. 1994). Thermal cycling conditions were 98 °C for 10 s, followed by 35 cycles of 94 °C for 1 min, 50 °C for 1 min, 72 °C for 1–2 min, and a final extension of 72 °C for 7 min, following the TaKaRa Ex manufacturer’s protocol. The amplified PCR products were purified and sequenced by Sangon Biotech (Shanghai). The PCR product size for the COI amplicon was 680 bp. The sequences obtained in this study have been uploaded to GenBank.

### DNA barcode dataset construction

We constructed a mitochondrial COI dataset with the newly obtained sequences from this study and the available *Inversidensbrandtii* sequences from GenBank.

Previously published sequences were downloaded from GenBank and added to the dataset, i.e., 17 species of the subfamily Gonideinae and four species of the subfamily Unioninae for the ingroup, and one species of the subfamily Parreysiinae as the outgroup.

As a result, a total of 29 COI sequences were used for this study. Sequence details and GenBank accession numbers are shown in Table 1.

**Table 1.** List of sequences used in this study. (*) Sequenced from this study.

Taxa	GenBank accession number
**UNIONIDAE Rafinesque, 1820**	
**Parreysiinae Henderson, 1935**	
*Indonaiaandersoniana* (Nevill, 1877)	KX865835
**Unioninae Rafinesque, 1820**	
*Acuticostachinensis* (Lea, 1868)	MG462919
*Inversiuniojokohamensis* (Ihering, 1893)	LC518985
*Inversiunioreinianus* (Kobelt, 1879)	LC518976
*Nodulariadouglasiae* (Griffith & Pidgeon, 1833)	NC_026111
**Gonideinae Ortmann, 1916**	
*Pseudodonbogani* Bolotov, Kondakov & Konopleva in Bolotov et al. 2017	MF352216
*Pseudodonmanueli* Konopleva, Kondakov & Vikrev in Bolotov et al. 2017	MF352228
*Monodontinacambodjensis* (Petit de la Saussaye, 1865)	KP795028
*Pilsbryoconchaexilis* (Lea, 1838)	KP795024
*Chamberlainiahainesiana* (Lea, 1856)	KX822635
*Sinohyriopsiscumingii* (Lea, 1852)	NC_011763
*Sinohyriopsisschlegelii* (Martens, 1861)	NC_015110
*Lamprotulacaveata* (Heude, 1877)	KX822646
*Lamprotulaleaii* (Griffith & Pidgeon, 1833)	NC_023346
*Potomidalittoralis* (Cuvier, 1798)	JN243905
*Pronodulariajapanensis* (Lea, 1859)	KX822659
*Gonideaangulata* (Lea, 1838)	DQ272371
*Leguminaiawheatleyi* (Lea, 1862)	KX822651
*Microcondylaeabonellii* (Férussac, 1827)	KX822652
*Sinosolenaiacarinata* (Heude, 1877)	KX822669
*Ptychorhynchuspfisteri* (Heude, 1874)	KY067440
*Parvasolenaiarivularis* (Heude, 1877)	KX966393
*Inversidensbrandtii* (Kobelt, 1879)	AB040827
*Inversidensbrandtii* (Kobelt, 1879)	MT020598
*Inversidensbrandtii* (Kobelt, 1879)	MT020597
*Inversidensbrandtii* (Kobelt, 1879)	LC519005
*Inversidensbrandtii* (Kobelt, 1879)	LC519004
*Inversidensrentianensis* sp. nov. 1*	MZ073336
*Inversidensrentianensis* sp. nov. 2*	MZ073337

All COI nucleotide sequences were translated to amino acid sequences using MEGA 5.0 (Tamura et al. 2011) and aligned based on the amino acid sequences using the program MUSCLE (Edgar 2004) with default settings. We calculated and compared inter-and intraspecific distances with MEGA 5.0 using the uncorrected *p*-distance. Standard error was assessed using 1000 bootstrap replicates.

### Phylogenetic analysis

Bayesian inference (BI) analyses were inferred in MrBayes Version 2.01 (Ronquist et al. 2012), using GTRGAMMAI model of nucleotide substitution. Four chains were run simultaneously for 10 million generations and trees were sampled every 1000 generations. The first 25% of these trees were discarded as burn-in when computing the consensus tree (50% Majority Rule). Sufficient mixing of the chains was considered to have been reached when the average standard deviation of split frequencies was below 0.01. Additionally, IQ-TREE was run for Maximum Likelihood (ML) tree reconstruction, using partition models with 1000 ultrafast bootstraps (Minh et al. 2013).

## Taxonomy

### Inversidens rentianensis

Taxon classificationAnimaliaUnionidaUnionidae

Wu & Wu sp. nov.

850559C4-00E4-5B14-8E80-2534213EEE3E

http://zoobank.org/62424717-9514-4C7D-9C0E-240F1D95F03E

Fig. 1C

#### Type specimens.

***Holotype*.** China • Jiangxi Province, Ganzhou City, Rentian Town (壬田镇), Mianshui River (25.989557°N, 116.131333°E), 13 March 2018, coll. Xiongjun Liu (NCFM180325). ***Paratypes*.** Same data as holotype (NCFM180326-NCFM180328).

#### Diagnosis.

*Inversidensrentianensis* sp. nov. is morphologically distinct from the other two recognized species within the genus by shell shape, beak position and nacre colour (Table 2). Diagnostic characteristics: shell irregularly subtriangular; curvature of the ventral margin slight, nearly straight; umbo situated 1/2 of shell length; nacre reddish.

**Table 2.** Conchological characters of *Inversidensrentianensis* sp. nov., *Inversidensbrandtii*, *Inversidenspantoensis*. Characteristic descriptions of *I.brandtii* and *I.pantoensis* are referenced from Kondo (1982, 2008) and He and Zhuang (2013).

	*I.rentianensis* sp. nov.	* I. brandtii *	* I. pantoensis *
Shell shape	Irregularly subtriangular	Ovate	Inequilateral, quadrate
Umbo position	1/2 of shell length	1/4 of shell length	1/3 of shell length
Umbo sculpture	Feebly wavy wrinkles	Rippled	Angularly wrinkled
Surface sculpture	Concentric ridges	Concentric ridges	Irregular growth lines
Nacre colour	Reddish	Milk-white	Bluish
Posterior slope	Sharp	Blunt	Blunt
Ventral margin	Nearly straight	Arc-shaped	Long and straight

#### Description.

Shell irregularly subtriangular, medium thickness, and quite inflated. Anterior margin regularly rounded; ventral margin nearly straight; posterior margin obliquely arc-shaped. Umbo prominent and slightly eroded. Umbo sculptured with feebly wavy wrinkles. Posterior slope formed by the ventral margin and posterior margin low, triangular. Epidermis shining black or with brownish-yellow hue. Only one cardinal tooth in each valve, shape triangular. Laterals thick, a little curved, 2 in each valve. Nacre reddish-bronze in colour.

Length 43–52 mm, height 29–36 mm.

#### Etymology.

The specific epithet is derived from the type locality, Rentian Town.

#### Distribution.

**Figure 2.**
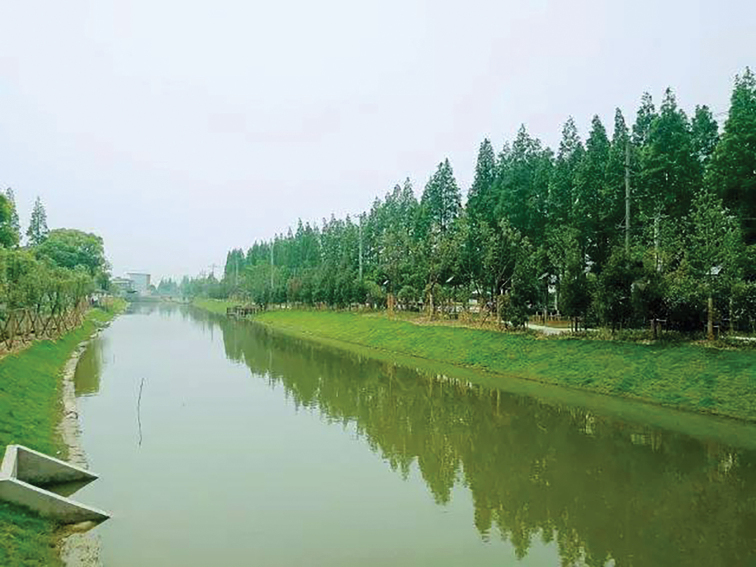
Photograph of sampling site of *Inversidensrentianensis* sp. nov. in China.

The species is known only from Mianshui River, Rentian Town, Ganzhou City, Jiangxi Province, China (present study) (Fig. 2).

#### GenBank accession number.

Holotype, NCFM180325: MZ073336; paratypes, NCFM180326: MZ073337.

#### Molecular analyses.

**Figure 3.**
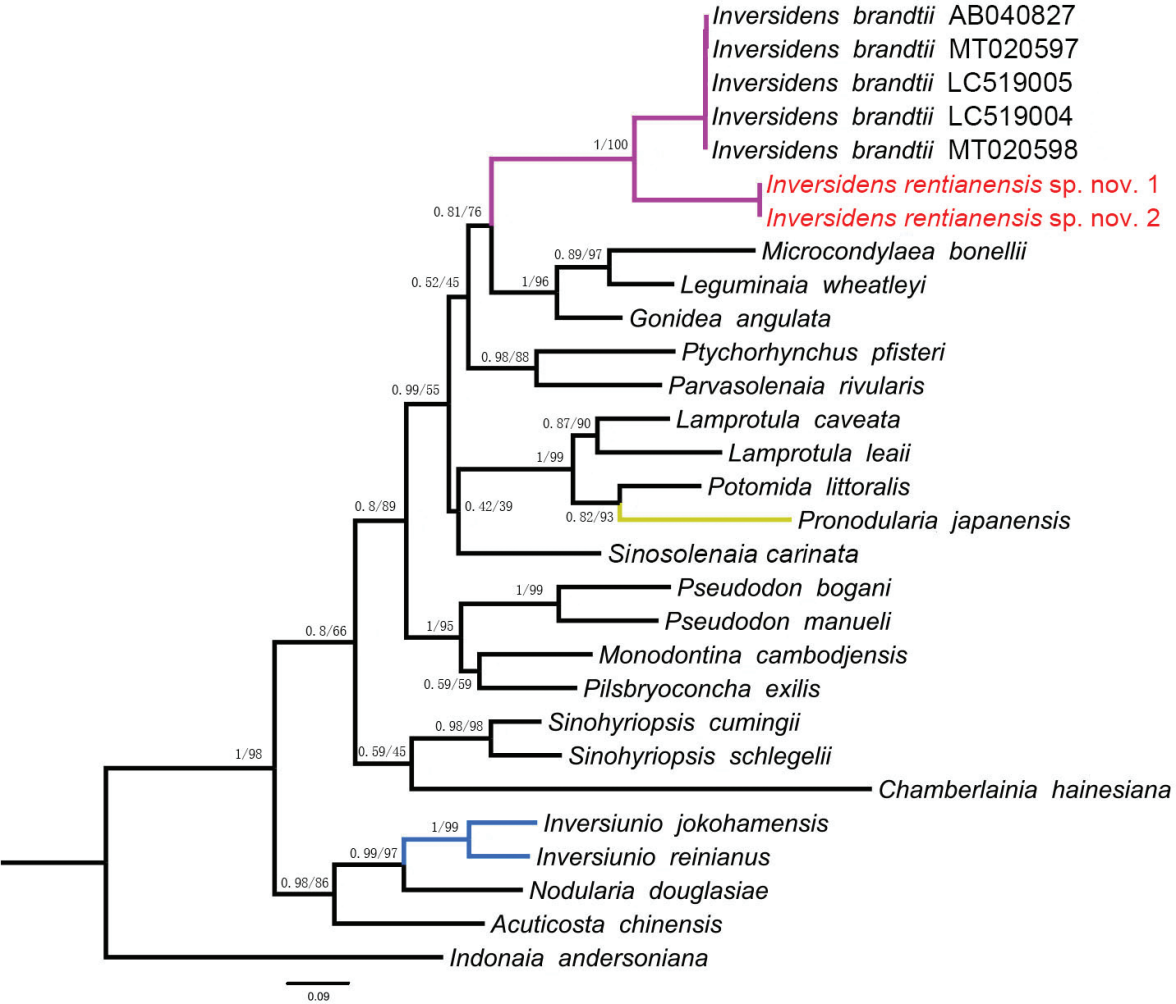
Phylogenetic tree of freshwater mussels inferred from Bayesian Inference (BI) and Maximum Likelihood (ML) analyses of COI barcode. Support values above the branches are posterior probabilities (PP)/bootstrap support (BS). Red font indicates the new species from this study.

Pairwise COI sequence divergences from *Inversidensbrandtii* and *Inversidensrentianensis* sp. nov. were conducted using MEGA 5.0. Based on the uncorrected *p*-distance model, the intraspecific divergences of *I.brandtii* and *I.rentianensis* sp. nov. were both 0.00%. The interspecific divergence of *I.brandtii* and *I.rentianensis* sp. nov. was 10.1%. Both BI and ML trees obtained a completely consistent topology. Consistent topology relationships are shown in Figure 3. In the phylogenetic trees, *I.rentianensis* sp. nov. formed a well-supported sister-group relationship with *Inversidensbrandtii* (PP = 1.00, BS = 100; Fig. 3). The genera *Pronodularia* and *Inversiunio* belong to different clades well-separated from *Inversidens* (Fig. 3).

#### Remarks.

Species delineation can be problematic in the presence of morphological ambiguities due to phenotypic plasticity and convergence (e.g., cryptic species), especially in mollusks (Zieritz et al. 2010; Inoue et al. 2013). The use of molecular genetics can aid species delineation in the case of phenotypic plasticity and/or convergence (Pieri et al. 2018; Wu et al. 2018). *Inversidensrentianensis* sp. nov. can be distinguished from congeneric species based on diagnostic characteristics of the shell. In this study, we also analyzed the interspecific divergence between *Inversidensbrandtii* and *Inversidensrentianensis* sp. nov. based on the COI barcode. The results showed that the average interspecific divergence between the two species was 10.1%, which was much higher than intraspecific divergences. Genetic analysis conducted in this study supports *I.rentianensis* sp. nov. as a valid species, which can be easily distinguished by the COI barcode.

## Supplementary Material

XML Treatment for Inversidens rentianensis
